# High-throughput detection of aberrant imprint methylation in the ovarian cancer by the bisulphite PCR-Luminex method

**DOI:** 10.1186/1755-8794-5-8

**Published:** 2012-03-26

**Authors:** Hitoshi Hiura, Hiroaki Okae, Hisato Kobayash, Naoko Miyauchi, Fumi Sato, Akiko Sato, Fumihiko Suzuki, Satoru Nagase, Junichi Sugawara, Kunihiko Nakai, Nobuo Yaegashi, Takahiro Arima

**Affiliations:** 1 Department of Informative Genetics, Environment and Genome Research Center, Tohoku University Graduate School of Medicine, 2-1 Seiryo-cho, Aoba-ku, Sendai 980-8575, Japan; 2 Department of BioScience, Tokyo University of Agriculture, 1-1-1 Sakuragaoka, Setagaya-ku, Tokyo 156-8502, Japan; 3 Departments of Obstetrics and Gynecology, Tohoku University Graduate, School of Medicine, Sendai, Japan; 4 Department of Development and Environmental Medicine, Tohoku University Graduate School of Medicine, Sendai, Japan

**Keywords:** Genomic imprinting, Ovarian cancer, DNA methylation, Bisulphite PCR-Luminex(BPL)method, LOI (loss of imprinting)

## Abstract

**Background:**

Aberrant DNA methylation leads to loss of heterozygosity (LOH) or loss of imprinting (LOI) as the first hit during human carcinogenesis. Recently we developed a new high-throughput, high-resolution DNA methylation analysis method, bisulphite PCR-Luminex (BPL), using sperm DNA and demonstrated the effectiveness of this novel approach in rapidly identifying methylation errors.

**Results:**

In the current study, we applied the BPL method to the analysis of DNA methylation for identification of prognostic panels of DNA methylation cancer biomarkers of imprinted genes. We found that the BPL method precisely quantified the methylation status of specific DNA regions in somatic cells. We found a higher frequency of LOI than LOH. LOI at *IGF2*, *PEG1 *and *H19 *were frequent alterations, with a tendency to show a more hypermethylated state. We detected changes in DNA methylation as an early event in ovarian cancer. The degree of LOI (LOH) was associated with altered DNA methylation at *IGF2/H19 *and *PEG1*.

**Conclusions:**

The relative ease of BPL method provides a practical method for use within a clinical setting. We suggest that DNA methylation of *H19 *and *PEG1 *differentially methylated regions (DMRs) may provide novel biomarkers useful for screening, diagnosis and, potentially, for improving the clinical management of women with human ovarian cancer.

## Background

Human ovarian cancer (HOC) is the leading cause of death from gynecological malignancies, primarily due to the lateness of detection when the cancer is already at an advanced stage. Effective screening protocols for early stages are not currently available. HOC is characterized by complex genetic and epigenetic alterations, including loss of heterozygosity (LOH) and loss of imprinting (LOI) [1,2]. Such alterations are presumed to represent the second hit, according to Knudson's two-hit hypothesis (OMIM #167000) [3]. However, alterations in DNA methylation can also occur as the first hit during human carcinogenesis [4].

For childhood cancers such as retinoblastoma (OMIM #180200), Wilms' tumor (OMIM #194070) and osteosarcoma (OMIM #259500), changes primarily occur on the paternal allele first, followed by a second hit on the maternal allele [5,6]. Complete hydatidiform moles, which are of androgenetic or paternal origin, are characterized by malignant transformation whereas ovarian teratomas, which are of parthenogenetic or maternal origin, are benign [7,8]. These observations suggest a role for altered genomic imprinting in the malignant transformation process.

Alterations in the expression of imprinted genes represent one of the most common changes seen in cancer [9,10]. Some imprinted genes, including *H19 *[11], *GTL2 *[12], *PEG1, PEG3 *[13], *LIT1 *(*KCNQ1OT1*) [14] and *ZAC *[15], are known to act, or strongly implicated to act, as tumor suppressor genes (TSGs). The monoallelic expression of imprinted genes is reliant on epigenetic mechanisms, most notably DNA methylation, which initiates the imprinting process in the male and female germlines at discrete locations termed differentially methylated regions (DMRs) [16]. Imprinted domains generally contain several genes displaying allele-specific expression and these DMRs, which can be located over the promoter of a protein coding gene or the promoter of a functional non-coding RNA or within intergenic regions, are known to control imprinted gene expression within the domain, acting as imprinting centers or imprint control regions [17]. We recently developed a new high-throughput, high-resolution DNA methylation analysis method called bisulphite PCR-Luminex (BPL) for the rapid analysis of DNA methylation [18]. In this study, we applied this method to 21 HOC cell lines and 74 HOC tissues to efficiently and accurately determine the methylation status of DMRs at eight imprinted loci, six of which contained TSGs. To determine whether abnormal methylation of these DMRs acts as an indicator for potential LOH and/or LOI, we also examined the association between abnormal hypermethylation and LOI or LOH. We found a higher frequency of LOI than LOH. LOI at *IGF2*, *PEG1 *and *H19 *was a frequent alteration, with a tendency to show a more hypermethylated status. The degrees of LOI and altered DNA methylation were similar among histology, progression and tumor grades. This suggests that DNA methylation of the *H19 *and *PEG1 *DMRs may provide novel biomarkers useful for screening, diagnosis and, potentially, for improving the clinical management of women with HOC.

## Results

### Frequencies of the 8 imprinted gene profiles in HOC

We first determined whether the ovarian malignancies showed LOH by comparing the restriction fragment length polymorphism (RFLP) patterns of normal lymphocyte DNA and 74 matching primary HOC DNA samples. Samples where RFLPs were present in the lymphocyte DNA sample but absent or with an altered ratio in the tumor sample were considered to exhibit LOH in the regions of 8 imprinted genes (*H19*, *IGF2*, *KCNQ1*, *LIT1*, *GTL2*, *PEG1*, *PEG3 *and *NDN*). The average percentage of heterozygosity was 48.0% (16.2-58.5%). We found only 14 cases of LOH in the 8 imprinted genes in the 74 HOC samples we analysed (Table 1). The most frequent gene with LOH was *IGF2 *(9.0%, 3/33), followed by *PEG1 *(8.1%, 3/37) and *GTL2 *(7.1%, 3/42). LOH of *NDN *and *LOT1 *was not detected (0/31 and 0/12). The samples with LOH were not from the same cases (Additional file 1: Table 1).

**Table 1:** Frequencies of LOH, LOI and MOI in human ovarian cancers.

(A) Ovarian cancer tissues								
	**H19/** **R sal**	**IGF2/** **Apal**	**KCNQ1/** **Smal**	**LIT1/** **R sal**	**GTL2/** **T aal**	**PEG1/** **AFlll**	**PEG3/** **Mnll**	**NDN/** **Mbol**

Heterozygosity	58.5 (41/70)	47.1 (33/70)	55.5 (40/72)	16.2 (12/74)	58.3 (42/72)	50.0 (37/74)	34.2 (25/73)	41.8 (31/74)
LOH	4.8 (2/41)	9.0 (3/33)	5.0 (2/40)	0.0 (0/12)	7.1 (3/42)	8.1 (3/37)	4.0 (1/25)	0.0 (0/31)
LOI	29.2 (12/41)	45.4 (15/33)	12.5 (5/40)	16.6 (2/12)	23.8 (10/42)	45.9 (17/37)	8.0 (2/25)	6.4 (2/31)
MOI	56.0 (23/41)	33.3 (11/33)	77.5 (31/40)	83.3 (10/12)	66.6 (28/42)	45.9 (17/37)	84.0 (21/25)	93.5 (29/31)
ND	9.7 (4/41)	12.1 (4/33)	5.0 (2/40)	0.0 (0/12)	2.3 (1/42)	0.0 (0/37)	4.0 (1/25)	0.0 (0/31)
(B) Cell lines								

Heterozygosity	19.0 (4/21)	14.2 (3/21)	33.3 (7/21)	14.39 (3/21)	23.8 (5/21)	42.8 (9/21)	23.8 (5/21)	23.8 (5/21)
LOI	0/4	2/3	3/7	1/3	2/5	3/9	0/5	0/5
MOI	4/4	1/3	4/7	2/3	3/5	6/9	5/5	5/5

Percents of LOH (loss of heterozygosity), LOI (loss of imprinting) and MOI (maintenance of methylation) determined using RFLP analysis of 8 imprinted genes in 74 samples of ovarian cancers and 21 cell lines

We next performed RT-PCR and RFLP analysis to identify the samples of LOI without LOH. The frequency of LOI was higher than that of LOH for all 8 imprinted genes and we found a total of 46 cases of LOI (Table 1, Additional file 1: Table S1). The most frequent sites of LOI were *PEG1 *(45.9%, 17/37), *IGF2 *(45.4%, 15/33) and *H19 *(29.2%, 12/41). *NDN *had the lowest frequency. In 19 of the 46 cases, the abnormal gene expression pattern was apparent at two or more imprinted loci. A normal imprinting pattern, maintenance of imprint (MOI), was most frequent in *NDN *(93.5%, 29/31). ND (not determined) means no amplification of RT-PCR at 3 times in several samples, perhaps indicating low expression of the genes. In 9 of the 14 LOH cases, LOI was also found in at least one gene. In HOC cell lines, LOI was found in 2 of 3 informative cases for *IGF2*, and 3 of 9 cases for *PEG1*. We did not find any LOH or LOI in 7 normal ovarian surface tissues and 4 normal cell lines. We compared patients' ages, progression, histology and tumor grades with imprinted gene expression pattern profiles. Patients with LOI had a tendency to be younger than patients with LOH (mean ages for LOH and LOI: 55.0 ± 7.4 and 47.7 ± 6.9, respectively), but the difference was not statistically significant by ANOVA, and no other correlations were apparent.

### Analysis of the methylation status of DMRs in ovarian cancers by the BPL method

**Figure 1:**
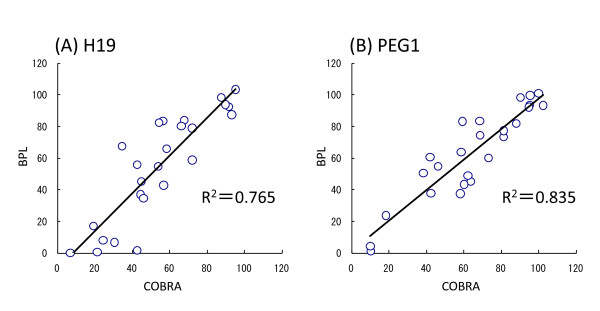
**Validation of BPL analysis by comparison with COBRA assay**. Examination of the imprinted DMRs by bisulphite PCR Luminex (BPL) and combined bisulphite PCR restriction analysis (COBRA) assay in DNA samples of ovarian cancer cell lines and normal cells. BPL: y-axis, COBRA: x-axis. Calculated by Spearman's rank method. *H19 *(A) and *PEG1 *(B).

The proof-of principle experiment of the BPL method has been described in detail [18]. Briefly, bisulphite-DNA can be used to distinguish between methylation and non-methylation status in the genome, e.g. cytosine and uracil. The BPL method can determine one base substitution by specific hybridization and detect the ratio of methylation to non-methylation. We examined the quality of the BPL method in spermatic DNA, which should show 100% methylation of the paternally methylated DMRs: *ZDBF2*, *H19 *and *GTL2*, whereas the maternally methylated DMRs: *PEG1, ZAC, SNRPN*, *PEG3 *and *LIT1 *are non-methylated. We applied the classic methylation assay COBRA technique and our recently devised BPL method to the DNA of 7 normal ovarian surface epithelium tissues, 4 primary cultures of normal human ovarian surface epithelium (OSE1-4) and 21 HOC cell lines, and performed statistical analysis with Spearman's and Pearson's rank correlations. For all 8 DMRs a good correlation was found between these two methods (Figure 1, Table 2, Additional file 2: Figure S1).

**Table 2:** The methylation profiles of 8 imprinted genes by bisulphite PCR Luminex.

Cell	Histology	H19		GTL2		ZDBF2		PEG1		LIT1		ZAC		PEG3		SNRPN	
		
lines		BPL	COBRA	BPL	COBRA	BPL	COBRA	BPL	COBRA	BPL	COBRA	BPL	COBRA	BPL	COBRA	BPL	COBRA
		
		CpG (9,16)	CpG10	CpG (4,8)	CpG8	CpG (1,4,5)	CpG4	CpG15	CpG12	CpG (5,17,19)	CpG16	CpG8	CpG7	CpG20	CpG21	CpG19	CpG19
NC1	N	67.5	34.6	62.2	76.8	66.4	46.2	50.4	38.5	**42.7**	**27.4**	62.3	48.4	78.8	67.4	65.3	57.3
NC2	N	55.6	42.7	**67.3**	**82.1**	67.9	72.5	54.8	46.2	53.7	37.5	65.2	69.3	65.4	48.9	78.5	67.3
NC3	N	**65.8**	**58.3**	81.2	63.5	76.3	82.1	**45.3**	**63.9**	26.4	41.6	50.2	72.1	65.8	83.2	56.3	68.2
NC4	N	58.6	72.1	76.4	80.4	80.2	67.2	37.5	58.2	**35.8**	**54.7**	47.2	52.8	45.9	58.3	65.2	74.9
OC1	S	**10.32**	**95.4**	69.8	53.2	87.3	92.4	**83.1**	**59.2**	64.3	48.3	89.5	98.3	74.4	83.2	65.4	58.4
OC2	M	16.8	19.4	61.4	70.5	95.7	97.2	48.8	62.2	62.1	48.0	20.1	10.2	**92.1**	**89.5**	29.1	49.7
OC3	S	87.4	93.2	96.3	93.4	83.7	28.6	**98.3**	**90.4**	87.4	56.9	87.3	96.5	98.5	96.4	28.4	37.4
OC4	S	42.8	56.8	97.3	93.2	99.3	98.5	0.9	10.4	13.2	17.1	100.9	98.3	**48.7**	**47.2**	28.1	24.2
OC5	S	83.4	56.4	89.2	96.6	95.3	72.8	93.2	102.4	28.4	32.6	100.3	91.3	98.8	95.6	54.3	60.4
OC6	E	78.9	72.1	94.3	82.1	38.3	18.3	83.2	68.5	**34.2**	**56.2**	85.3	78.9	57.2	97.5	40.5	53.1
OC7	C	0.4	21.3	19.8	29.3	41.7	55.5	60.5	42.0	21.2	11.3	34.5	40.1	90.7	99.0	23.5	52.8
OC8	C	0.1	6.9	6.4	10.5	91.7	87.3	**81.8**	**88.1**	35.2	29.1	49.8	53.2	102.1	95.7	21.8	33.2
OC9	S	82.3	54.3	**37.4**	**65.2**	22.4	42.8	**23.6**	**18.5**	43.7	56.8	69.5	74.9	**68.5**	**90.2**	38.2	56.3
OC10	C	83.8	67.8	**78.4**	**65.9**	27.9	32.3	63.8	58.7	42.8	60.2	89.9	93.7	59.6	43.9	38.9	71.4
OC11	S	92.4	91.6	17.1	25.0	6.0	18.0	43.2	60.1	18.2	10.0	72.5	58.2	101.4	100.0	75.5	60.5
OC12	S	54.7	53.8	23.7	18.8	48.6	**74.3**	**68.9**	58.4	48.3	93.6	98.8	**67.4**	**39.8**	**73.4**	57.1	51.2
OC13	S	**80.3**	**66.2**	**42.3**	**40.1**	86.0	74.2	**93.4**	95.2	10.5	8.8	94.4	90.5	**102.1**	**100.0**	36.0	39.5
OC14	C	98.1	87.7	67.4	40.3	99.3	90.2	99.6	95.5	12.3	1.6	34.3	41.2	92.1	100.0	38.1	51.2
OC15	C	45.2	45.0	93.0	98.2	99.7	95.3	**92.1**	**94.8**	55.0	47.5	27.5	36.4	86.1	97.2	48.7	10.1
OC16	S	36.9	44.6	90.6	92.0	93.0	92.3	100.7	100.0	13.0	6.2	28.9	35.7	93.2	100.0	22.9	17.6
OC17	C	93.8	89.8	61.8	68.3	21.8	22.2	**37.9**	**42.5**	**48.4**	**31.8**	20.6	31.1	98.3	95.5	0.0	0.2
OC18	C	**34.4**	**46.0**	94.5	98.0	95.3	98.0	60.1	73.2	35.2	29.4	17.3	35.0	87.2	100.0	22.7	11.1
OC19	C	67	30.7	**47.8**	**62.1**	87.4	85.3	4.1	10.1	**11.2**	**0.0**	100.1	100.0	84.6	92.1	30.4	31.4
OC20	M	1.6	42.6	**58.1**	**60.3**	62.4	55.2	**73.4**	**81.4**	18.3	13.4	49.2	68.2	92.8	98.5	86.7	44.8
OC21	S	**8.0**	**24.5**	**80.2**	**87.0**	96.6	90.1	77.2	81.2	4.5	8.1	100.5	97.5	107.8	98.2	16.7	16.2

*Normal ovarian surface tissues*																	
NT1 (42)		57.5	76.2	43.4	51.2	37.2	65.4	51.4	38.6	**49.1**	**42.3**	53.3	41.2	42.1	40.2	521	47.8
NT2 (48)		**27.6**	**25.0**	23.6	21.6	46.1	34.5	20.9	34.4	23.1	12.2	5.1	32.3	21.3	20.6	22.1	33.3
NT3 (40)		61.5	31.8	**49.9**	**60.0**	50.4	45.0	31.0	42.8	17.1	10.9	48.8	29.4	21.0	33.6	24.3	34.8
NT4 (38)		28.9	25.0	**28.4**	**29.6**	46.1	39.3	**33.4**	**47.8**	28.8	17.7	67.4	46.1	45.6	33.8	80.5	29.0
NT5 (55)		**24.2**	**26.9**	**27.2**	**18.5**	33.2	29.0	**30.9**	**48.1**	**25.1**	**17.6**	10.7	44.5	75.5	40.0	32.3	31.3
NT6 (45)		**25.9**	**22.1**	**32.2**	**20.5**	46.4	62.5	60.0	40.4	24.1	17.3	25.2	23.4	44.2	69.0	77.4	40.9
NT7 (41)		34.6	24.6	**38.0**	**19.9**	49.9	37.4	27.3	46.2	**27.7**	**24.5**	21.0	63.1	49.7	53.7	25.6	24.5

Letters in blue and black boldface represent LOH, LOI and MOI, respectively. The Luminex value indicates the average value of DNA methylation

We next determined the methylation status of the 8 DMRs from the 74 samples of primary ovarian cancer tissue by the BPL method. Overall, we compared the average DNA methylation status of cancer and normal samples for each DMR and found that *PEG1 *from ovarian cancers was significantly more hypermethylated than normal ovarian tissues (normal, 30.7% ± 15.1: HOC, 45.9% ± 15.5) (Table 3). The numbers of cancer tissue cases with hypermethylation above the range of the methylation rates in the normal ovarian surface epithelium were 17 for *H19*, 21 for *GTL2*, 21 for *ZDBF2*, and 14 for *PEG1 *(Table 3). On the other hand, hypomethylation below the methylation level of normal ovarian tissues was found in 15 cases for *GTL2 *and 23 for *ZDBF2*. We did not observe a significant difference in the DNA methylation between localized early-stage and advanced-stage tumor groups (Table 3). This suggested that the DNA methylation changes we detected occurred as early events of ovarian cancer.

**Table 3:** Characterization of methylation profiles of the imprinted genes in DNA of ovarian cancers.

(A) Histology									
		**H19**	**GTL2**	**ZDBF2**	**PEG1**	**LIT1**	**ZAC**	**PEG3**	**SNRPN**

Normal	(n = 7)	34.3 ± 11.8	34.7 ± 9.5	44.2 ± 6.6	30.7 ± 15.1	27.9 ± 12.6	33.1 ± 5.8	42.8 ± 18.5	44.9 ± 25.3
Cancer	(n = 74)	41.7 ± 17.2	39.6 ± 18.8	43.8 ± 6.6	45.9 ± 15.5*	27.9 ± 14.1	41.1 ± 6.4	41.1 ± 14.1	42.8 ± 12.9
Serous	(n = 36)	47.6 ± 18.9	38.8 ± 22.0	42.0 ± 18.5	48.9 ± 14.7*	28.8 ± 9.8	42.7 ± 16.6	40.9 ± 14.8	39.3 ± 12.2
Mucinous	(n = 9)	36.0 ± 15.0	35.2 ± 19.2	36.8 ± 14.0	47.1 ± 20.0	34.7 ± 21.4	42.2 ± 5.9	31.2 ± 11.2	42.7 ± 17.8
Endometrioid	(n = 10)	37.9 ± 14.8	30.7 ± 14.4**	57.8 ± 19.8	45.5 ± 13.7	22.8 ± 13.0	37.5 ± 11.5	46.8 ± 15.9	41.4 ± 10.8
Clear	(n = 18)	45.6 ± 20.2	54.0 ± 20.1	39.7 ± 20.8	42.2 ± 13.8	25.5 ± 12.4	40.2 ± 16.1	45.7 ± 14.5	47.8 ± 11.1
(B) Progress (Staging)									

Localized (I, II)	(n = 29)	38.8 ± 14.9	40.8 ± 20.4	46.7 ± 19.4	47.8 ± 17.1	30.4 ± 16.1	41.0 ± 11.9	42.8 ± 17.8	44.2 ± 12.9
Advanced (III, IV)	(n = 45)	47.8 ± 19.6	41.3 ± 22.2	41.9 ± 19.2	45.9 ± 13.7	26.2 ± 10.0	41.1 ± 16.8	41.2 ± 12.8	40.9 ± 12.6
(C) Age									

Under 44 years	(n = 17)	46.7 ± 18.5	35.3 ± 21.0	42.0 ± 20.4	48.9 ± 13.5	24.2 ± 11.7	39.9 ± 12.3	44.7 ± 14.3	36.3 ± 10.7
45-55 years	(n = 29)	39.9 ± 14.7	47.2 ± 23.0	45.8 ± 17.5	46.9 ± 15.0	30.1 ± 14.8	38.9 ± 15.5	41.9 ± 15.8	45.4 ± 11.1
Over 56 years	(n = 32)	47.4 ± 21.2	38.4 ± 18.1	42.2 ± 21.1	45.1 ± 16.2	28.2 ± 10.9	44.1 ± 15.7	40.8 ± 14.9	42.5 ± 14.5

The values in the list are mean ± SD (standard deviation). Statistically significant differences between groups are presented as *P < 0.05, and **P < 0.01 by ANOVA

### The association between DNA methylation status and LOH/LOI in ovarian cancers

To determine whether the DNA methylation status in these DMRs of the imprinted genes acts as an indicator for potential LOH and/or LOI, we evaluated the association between DNA methylation and LOH and/or LOI in the *IGF2/H19 *and *PEG1 *imprinted domains separately.

**Figure 2:**
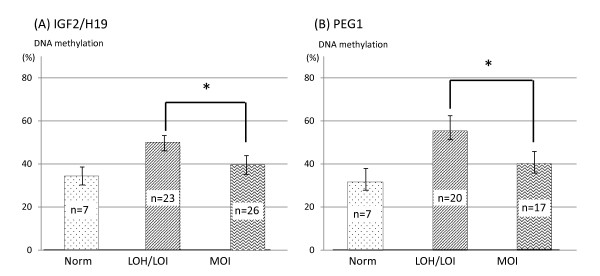
**The association between hypermethylation and LOH/LOI**. (A) *IGF2/H19 *DMR. The rate of DNA methylation (mean ± SD) of cases with LOH/LOI of either *IGF2 *or *H19 *and MOI were compared. For this analysis, one case which *H19 *was MOI and *IGF2 *was LOI, was removed. (B) *PEG1 *DMR. Statistically significant differences between groups are analyzed with ANOVA followed with multiple comparison method (Tukey's HSD test), presented as the *P*-value (*P < 0.01). Norm: Normal ovarian tissues.

*IGF2*, which acts as a dominant oncogene, and *H19*, a physically and mechanistically linked gene on human chromosome 11, are reciprocally imprinted. In the paternal allele, *H19 *DMR is methylated and silenced, whereas the reciprocally imprinted gene *IGF2 *is transcribed. By contrast, in the maternal unmethylated allele, *H19 *is expressed but *IGF2 *is inactivated because of the binding of the repressor factor CTCF to the unmethylated *H19 *DMR, which then prevents the *H19/IGF2 *common enhancers from activating the *IGF2 *promoter [19]. *IGF2 *was found to have high frequencies of both LOH and LOI in HOC. *H19 *was also found to have high frequencies of LOI (29.2%, 12/41). Nine of 14 cases with both *IGF2 *and *H19 *heterozygosity showed LOH or LOI of both genes and only one case had MOI for one of the two. Thus relaxation of *IGF2 *and *H19 *imprinting is frequent. In the *IGF2/H19 *imprinted region, the samples with LOH and/or LOI at *H19 *was more methylated than those with MOI (Figure 2A, Additional file 3: Table S2). Our results for *H19 *were similar to a previously reported finding [20]. *PEG1 *was reported to be a TSG and was also found to have high frequency of LOH/LOI in HOC. We also found that the samples with LOH/LOI at *PEG1 *were more methylated than those with MOI with statistical significance (Figure 2B, Additional file 3: Table S2).

## Discussion

Alterations in DNA methylation are the most common molecular alterations in human malignancies. Detection of the aberrant DNA methylation associated with cancer-related genes is a promising approach to improve cancer prevention, diagnosis and treatment options. Bisulphite modification is a prerequisite for most popular techniques aiming at detecting changes in methylation, but has been limited by throughput capacity. In this study, we used a high-throughput methylation detection method to analyze DNA methylation at 8 imprinted DMRs in epithelial ovarian cancer. We found that the PCR-Luminex method precisely quantified the methylation status of specific DNA regions in somatic cells and was also relatively rapid, economical and easy to use.

In the epithelial ovarian cancers, the frequency of LOI was higher than that of LOH. In particular, LOI was most frequent at *PEG1*, *IGF2 *and *H19 *DMRs. The frequency, extent of changes in DNA methylation and loci affected varied considerably among the samples. Generally, we found that DNA methylation at imprinted DMRs was increased in both cell lines and primary material. Importantly, we showed that gain of DNA methylation in the imprinted DMRs was apparent in tumors with LOI, especially at *PEG1 *and *H19*. We also found DNA methylation changes in the absence of LOI. In other words, there were changes in DNA methylation at DMRs that were not associated with biallelic gene expression.

When we examined the clinical characteristics of the tumors, we found no significant differences in the frequency of LOI and aberrant DNA methylation between the localized early-stage and advanced-stage tumor groups. This suggested that the changes we identified occurred as a relatively early event of HOC. In general, the *PEG1 *and *H19 *DMRs appeared to be particularly prone to errors. This is similar to the previous findings in human sperm from subfertile men [21]. In *ZDBF2 *and *GTL2 *DMRs, aberrant DNA methylation occurred in HOC. As with *H19*, these DMRs are paternally methylated DMRs in somatic cells. In childhood cancers such as retinoblastoma, Wilms' tumor and osteosarcoma, changes primarily occur on the paternal allele first, followed by a second hit on the maternal allele [5,6,22]. Similarly, methylation of paternally imprinted DMR in normal somatic cells might be a first hit and cause ovarian carcinogenesis. These observations suggest a role for altered genomic imprinting in the malignant transformation process.

A previous report had demonstrated the association between the abnormal genomic imprinting of *H19 *and *IGF2 *expression [20]. The aberrant hypermethylation in the CTCF binding site of the *H19 *gene was seen in the cases of HOC and correlated with *IGF2 *LOI. Our results for *H19 *were similar to those reported findings. The most frequent methylation error in HOC was seen in the *PEG1 *DMR. In our previous report, we showed that demethylation of *PEG1 *was present in growing oocytes from superovulated infertile women [23]. This *PEG1 *DMR may be especially vulnerable to errors. LOI of PEG1 has subsequently also been implicated in the aetiology of lung adenocarcinomas, breast and colon cancer.

HOC is the leading cause of death from gynecologic malignancies because the majority of cases are not detected until the disease is well advanced. Our understanding of cancer as a clonal genetic disease has led to the identification of genetic alterations in many cancer types. However, ovarian cancer remains less well characterized. Only a few TSG genes acting in a recessive manner have been identified as somatically mutated or methylated in ovarian cancer, including *TP53 *(48%) [24], *PTEN *(21% in the endometrioid subtype) [25], *RB1 *(7-10%) [26], and *CDKN2A *(79% in the mucinous subtype) [27]. Biomarkers provide useful tools in screening for cancer and are now emerging as highly informative for monitoring disease status [28]. They can improve early detection and also the quality of life of patients with ovarian cancer. DNA methylation offers an additional tool that can be used in combination with other markers [29]. In addition, it has been established that DNA methylation biomarkers are present in patient serum and other body fluids [30]. To date, several methylated genes have been found to be highly prognostic for specific cancers, including those of the prostate [31], breast [32] and lung [33]. Although some methylated markers such as RASSF1A and GSTP1 have potential as prognostic indicators individually [34,35], 'methylation signature' panels could be much more informative [36] and accurate for monitoring cancer progression. Methylation patterns have previously been suggested to be tumor and stage specific [37]. Our work demonstrates that there is aberrant DNA methylation at several imprinted DMRs in HOC with changes at *PEG1 *and *H19 *being the most frequent and earliest alterations detected.

## Conclusion

This is the first study reporting the use of PCR-Luminex for identification of prognostic panels of DNA methylation biomarkers for cancer. We believe that this approach is amenable to the classification of clinically relevant methylation patterns in a wide variety of tumors (and other pathologies) linked to the aberrant DNA methylation of imprinted DMRs. This BPL method may be sufficiently sensitive that it can be applied to the analysis of DNA methylation in the very small number of circulating cancer cells found in blood and urine samples from patients.

## Methods

### Ovarian cancer cell lines and primary culture of surface epithelial cells

Twenty-one HOC cell lines were used in our study: 10 from serous adenocarcinoma (OVCAR3, CAOV3, JHOS2, HTOA, SKOV3, OV90, JHOS3, JHOS4, KF, MH), 2 from mucinous adenocarcinoma (OMC3, MCAS), 8 from clear cell adenocarcinoma (ES2, JHOC5, TOV21G, JHOC7, JHOC8, KM, HAC2, RMG) and 1 from endometrioid adenocarcinoma (TOV112D). The sources of these cells and culture methods were as described previously [38,39]. Four primary cultures of normal human ovarian surface epithelial (OSE1-4) cells were initiated from surface scrapings of normal ovaries as described [40].

### Ovarian cancer tissue(s)

Seventy-four primary HOC tissues (36 serous, 9 mucinous, 10 endometrioid, 18 clear cell, and 1 other, Table 3) were obtained from patients presenting at our hospital. The mean ± standard deviation (SD) of the patients' ages for normal ovary and ovarian cancer tissues were 44.1 ± 6.6 and 52.9 ± 7.3, respectively. Seven specimens of normal ovarian surface epithelium were obtained from patients with benign non-ovarian disease. Histological diagnoses and clinical staging were performed according to the International Federation of Gynecologists and Obstetricians (FIGO) criteria. The numbers of cancer patients with localized tumors (stage I and II) and advanced tumor (stages III and IV) were 29 and 45, respectively. The samples were stained with hematoxylin and eosin to demonstrate > 85% of epithelial tumor cells. DNA and RNA were then extracted from the remaining samples [38]. DNA was also extracted from peripheral blood in matched patients. The study was performed after obtaining the patients' informed consent and with approval from the institutional ethics committee of the Tohoku University Graduate School of Medicine.

### Analysis of loss of heterozygosity (LOH) and loss of imprinting (LOI)

PCR was performed on patient blood and tumor genomic DNA using the primer sequences summarized in Additional file 4: Table S3. A PCR reaction mix containing 0.5 μM of each primer set, 200 μM dNTPs, 1 × PCR buffer, and 1.25U of EX *Taq *Hot Start DNA Polymerase (Takara Bio, Tokyo, Japan) in a total volume of 20 μl was used. The following PCR program was used: 1 minute of denaturation at 94°C followed by 35 cycles of 30 seconds at 94°C, 30 seconds at 60°C and 30 seconds at 72°C and a final extension for 5 minutes at 72°C. PCR products were digested by unique polymorphic enzymes to identify samples that were heterozygous for a single nucleotide polymorphism (SNP). For samples found to be heterozygous for a SNP, RNA was prepared from matched tumors, followed by reverse transcription-PCR (RT-PCR) and by restriction digestion [41-48]. The digested PCR products were electrophoresed on 2% agarose gel.

### DNA methylation analysis

Bisulphite PCR-Luminex (BPL) methylation analysis was performed as described [18]. PCR primers sets, biotinylated at their 5'-end, were designed for gene amplification. PCR reaction mix contained 0.2 μM primer, 0.2 mM dNTPs, 1 × PCR buffer (50 mM KCl, 10 mM Tris-HCl, pH 8.3). 3 mM MgCl_2_, 2% dimethyl sulfoxide (DMSO), 0.625U *Taq *DNA Polymerase (Roche, Tokyo, Japan) and 100-200 ng of bisulphite treated DNA in a total volume of 25 μl. PCR conditions: 40 cycles of 95°C for 20 s/60°C for 30 s/72°C for 30 s using a GeneAmp 9700 thermal cycler (Applied Biosystems, CA, USA). Oligonucleotide probe sequences (Additional file 2: Table S1 in Ref 18) were synthesized and covalently bound to carboxylated fluorescent microbeads using ethylene dichloride (EDC). These oligonucleotide-labeled microbeads (oligobeads) were mixed together to make an oligobeads mixture of 100 oligobeads/μl and hybridized to the 5'-biotin-labeled PCR amplicons in a total volume of 50 μl per well in a 96-well plate by adding 5 μl of the appropriate oligobead mixture and 5 μl of the PCR amplicons to 40 μl of hybridization buffer. This reaction mixture was first denatured at 95°C for 2 min and then hybridized at 48°C for 30 min. After hybridization, the oligobeads were washed in 100 μl of PBS-Tween and pelleted by microcentrifugation. Pelleted oligobeads were reacted with a 70 μl aliquot of a 100 × diluted solution of SA-PE in PBS-Tween. Hybridized amplicons were labeled with SA-PE at 48°C for 15 min. Reaction outcomes were measured by the Luminex 100 flow cytometer. Methylation assays were additionally performed for each DMR using the conventional bisulphite treatment PCR methylation assay and combined bisulphite PCR restriction analysis (COBRA) as described previously [21].

### Statistical analyses

Differences between groups were analysed by analysis of variance, followed by Post-hoc, Tukey's HSD test. Statistical analyses were performed using the JMP (v9.0.0, SAS Institute Japan, Tokyo, Japan). Statistically significant differences between groups are presented as *P < 0.05, and **P < 0.01. Results for BPL and COBRA were compared using Spearman's rank method and Pearson's product-moment correlation coefficients.

## Abbreviations

BPL: Bisulphite PCR-Luminex; COBRA: Combined bisulphite PCR restriction analysis; DMR: Differentially methylated region; FIGO: International Federation of Gynecologists and Obstetricians; HOC: Human ovarian cancer; LOH: Loss of heterozygosity; LOI: Loss of imprinting; MOI: Maintenance of imprint; ND: Not determined; PCR: Polymerase chain reaction; QOL: Quality of Life; RFLP: Restriction fragment length polymorphism; RT-PCR: Reverse transcription-PCR; SNP: Single nucleotide polymorphism.

## Competing interests

The authors declare that they have no competing interests.

## Authors' contributions

HH, HO, HK, NM, FS and AS performed the DNA methylation analyses and validation of the BML method. FS, SN, JS, NY carried out the product and culture of cancer cell lines and collect the tumor samples. KN did the statistical analyses. HH and TA wrote this manuscript. All authors have read and approved the final manuscript.

## Pre-publication history

The pre-publication history for this paper can be accessed here:

http://www.biomedcentral.com/1755-8794/5/8/prepub

## Supplementary Material

Additional file 1 **Table S1 The list of LOH, LOI and MOI in HOC**. LOH, LOI and MOI determined using RFLP analysis of 8 imprinted genes are summarized. NC: Normal cells (NC1-4). NT: Normal ovarian tissues (NT1-7). CC: Cancer cell lines (CC1-21). CT: Cancer tissue (CT1-74). Click here for file

Additional file 2 **Figure S1 Validation of BPL analyses by comparison with COBRA assay**. Examination of the imprinted DMRs by bisulphite PCR Luminex (BPL) and combined bisulphite PCR restriction analysis (COBRA) assay in DNA samples of ovarian cancer cell lines and normal cells. BPL: y-axis, COBRA: x-axis. The number was calculated by Spearman's rank method. *GTL2 *(C), *ZDBF2 *(D), *LIT1 *(E), *ZAC *(F), *PEG3 *(G) and *SNRPN *(H). Click here for file

Additional file 3 **Table S2 Sequences of PCR primers and restriction enzymes used for PCR-RFLP analysis**. Click here for file

Additional file 4 **Table S3 Bisulphite PCR-Luminex and COBRA methylation profiles of the eight imprinted DMRs in the DNA of human ovarian cancer cells and normal ovarian tissues**. Numbers in blue and black boldface indicate LOI and MOI, respectively. Luminex values indicate average methylation values at the sites tested. NC: Normal cells (OSE1, OSE2, OSE3, OSE4). CC: Cancer cell lines (CC1: OVCAR3, CC2: OMC3, CC3: CAOV3, CC4: JHOS2, CC5: HTOA, CC6: TOV112D, CC7: ES2, CC8: JHOC5, CC9: SKOV3, CC10: TOV21G, CC11: OV90, CC12: JHOS3, CC13: JHOS4, CC14: JHOC7, CC15: JHOC8, CC16: KF, CC17: KM, CC18: HAC, CC19: RMG, CC20: MCAS, CC21: MH. NT: Normal ovarian tissues (NT1-NT7). N: Normal, S: Serous adenocarcinoma, M: Mucinous adenocarcinoma, E: Endometrioid carcinoma, C: Clear cell carcinoma. Click here for file
