# Supplementary material for: High-throughput detection of aberrant imprint methylation in the ovarian cancer by the bisulphite PCR-Luminex method
Source: BMC Med Genomics. 2012 Mar 26;5:8. doi: 10.1186/1755-8794-5-8 (PMC3342152; doi:10.1186/1755-8794-5-8)
Supplement: Additional file 2 — Figure S1 Validation of BPL analyses by comparison with COBRA assay. Examination of the imprinted DMRs by bisulphite PCR Luminex (BPL) and combined bisulphite PCR restriction analysis (COBRA) assay in DNA samples of ovarian cancer cell lines and normal cells. BPL: y-axis, COBRA: x-axis. The number was calculated by Spearman's rank method. GTL2 (C), ZDBF2 (D), LIT1 (E), ZAC (F), PEG3 (G) and SNRPN (H). [file 1755-8794-5-8-S2.PPT]

## Slide 1
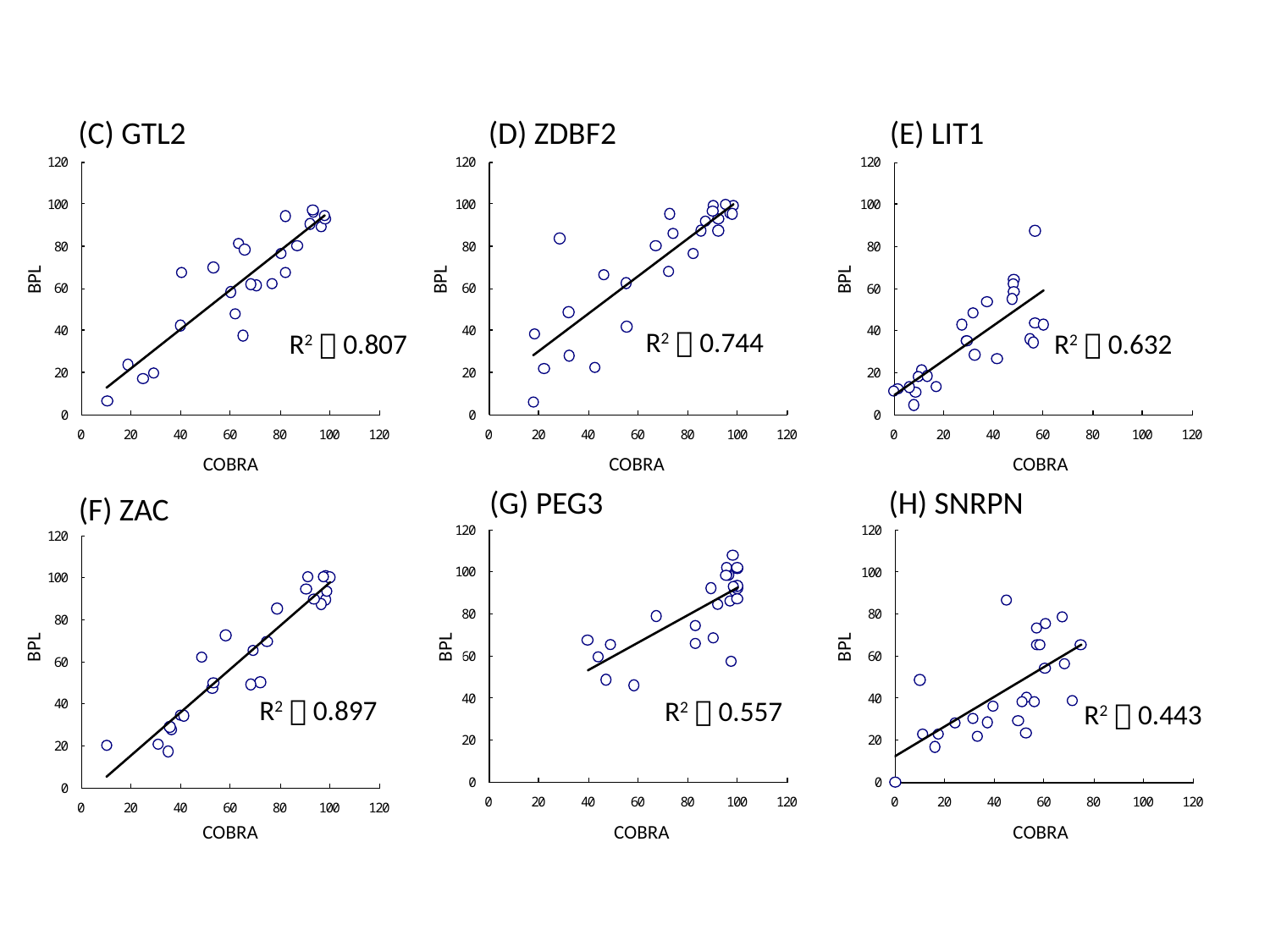

(C) GTL2
BPL
COBRA
R2＝0.807
(D) ZDBF2
BPL
COBRA
R2＝0.744
(E) LIT1
BPL
COBRA
R2＝0.632
(G) PEG3
BPL
COBRA
R2＝0.557
(H) SNRPN
BPL
COBRA
R2＝0.443
(F) ZAC
BPL
COBRA
R2＝0.897
