# Supplementary material for: High-throughput detection of aberrant imprint methylation in the ovarian cancer by the bisulphite PCR-Luminex method
Source: BMC Med Genomics. 2012 Mar 26;5:8. doi: 10.1186/1755-8794-5-8 (PMC3342152; doi:10.1186/1755-8794-5-8)
Supplement: Supplementary file 2 [file 1755-8794-5-8-S1.DOC]

Supplemental Table 1. The methylation profiles of 8 imprinted genes by bisulphite PCR Luminex.

Case	Age	Histology	Stage	Grade	H19	GTL2	ZDBF2	PEG1	LIT1	ZAC	PEG3	SNRPN
CT1	59	S	I c	2	61.7	**33.9**	39.6	69.5	31.9	43.0	11.4	37.1
CT2	63	C	III c	1	35.5	28.5	37.4	68.7	14.3	38.2	21.1	42.2
CT3	68	M	I c	1	**48.0**	**26.2**	38.4	57.3	**21.5**	49.3	14.1	50.2
CT4	46	S	III c	2	43.1	**66.9**	66.4	58.3	27.3	54.1	52.6	47.8
CT5	54	S	III b	1	29.8	84.5	51.6	52.3	27.4	35.3	54.2	53.9
CT6	55	C	I c	3	33.6	89.6	54.6	51.2	39.9	39.2	56.7	44.3
CT7	45	E	IV	1	26.6	**16.6**	30.6	54.8	15.5	11.4	26.7	40.8
CT8	48	M	III c	2	42.2	**46.9**	17.8	36.1	**32.8**	48.9	13.7	49.0
CT9	43	S	III c	3	39.4	**52.2**	36.9	65.3	21.4	35.3	**35.3**	38.2
CT10	45	S	I c	1	**46.4**	32.5	32.5	82.4	19.9	42.8	47.1	37.1
CT11	36	S	III c	1	**45.0**	**47.4**	5.6	**62.2**	19.2	69.0	49.7	19.3
CT12	59	S	III c	3	85.1	**29.4**	28.1	52.9	24.5	42.9	**45.9**	31.2
CT13	51	C	I c	1	**74.3**	28.3	80.7	**47.0**	44.4	24.7	**42.3**	34.7
CT14	39	S	III c	2	23.9	3.4	59.8	61.3	36.7	42.3	23.5	36.2
CT15	67	S	III c	2	77.0	4.4	27.1	**52.0**	17.5	42.5	52.3	16.3
CT16	43	E	II c	2	47.8	1.7	75.1	59.4	8.1	39.4	**51.4**	49.8
CT17	57	S	IV	1	**21.7**	**24.4**	17.2	**78.9**	32.8	55.7	**48.3**	27.6
CT18	64	E	IV	2	47.9	**31.7**	85.0	49.4	**24.2**	42.3	**40.6**	42.8
CT19	45	C	I c	1	**37.0**	**60.5**	27.6	**56.2**	**13.6**	49.7	**49.0**	48.8
CT20	41	M	I c	1	**21.5**	**34.6**	39.3	**64.2**	19.0	49.1	**39.4**	56.0
CT21	48	S	II	1	**62.9**	**89.9**	37.1	**33.8**	40.9	28.8	**29.4**	21.9
CT22	50	S	III c	3	32.7	**88.3**	37.6	**49.4**	17.2	21.3	**31.5**	44.0
CT23	41	S	III c	1	**44.7**	21.2	38.7	**60.2**	12.4	36.5	66.1	43.9
CT24	61	S	IV	3	**40.2**	**31.1**	64.2	55.7	29.5	57.9	51.4	25.4
CT25	52	C	I	1	45.4	**69.3**	46.7	**37.9**	11.5	81.3	**46.7**	53.8
CT26	49	S	III	1	70.6	11.8	37.7	**32.0**	25.1	42.9	21.5	42.3
CT27	68	S	IV	1	**62.1**	44.8	46.4	59.6	**11.2**	90.3	**59.9**	45.6
CT28	52	C	III	2	**50.8**	**83.8**	53.5	25.3	22.3	28.0	**43.8**	40.2
CT29	39	C	III	2	**71.8**	66.2	55.9	35.8	9.3	24.3	37.2	38.2
CT30	39	E	III	1	**26.1**	41.0	69.5	**32.4**	18.1	40.9	**23.4**	22.4
CT31	55	S	II	1	**32.9**	**34.9**	73.5	30.9	38.7	26.8	19.6	37.2
CT32	56	M	III	1	34.3	**29.8**	44.6	**26.7**	10.1	35.1	**41.7**	37.6
CT33	36	E	I	2	**34.4**	**42.1**	74.0	**51.5**	27.2	55.2	63.3	25.9
CT34	74	C	II	1	**32.8**	53.8	15.2	14.5	39.9	53.8	79.8	52.3
CT35	64	S	III	2	32.8	21.8	30.3	23.0	20.4	13.1	14.8	76.9
CT36	47	S	III	2	29.4	12.4	39.3	**56.9**	19.3	14.9	37.6	55.4
CT37	74	S	III	2	26.9	47.0	78.5	**66.1**	28.7	34.6	38.9	51.0
CT38	53	E	I	2	26.1	**42.0**	56.3	**50.0**	18.0	43.9	**39.7**	56.5
CT39	53	S	III	3	**64.8**	**44.5**	48.9	**35.4**	**40.4**	39.9	42.1	58.3
CT40	57	C	III	2	**60.8**	42.5	21.1	**42.1**	27.6	40.6	35.2	71.9
CT41	43	C	III	2	13.3	79.8	18.6	**57.8**	25.5	19.4	**70.9**	29.6
CT42	56	Other	III	3	**40.1**	**50.6**	38.5	62.2	20.6	57.3	47.0	48.4
CT43	51	E	I	2	19.2	**43.7**	72.6	**64.4**	17.4	23.2	75.6	52.4
CT44	56	C	I	2	**67.6**	52.7	12.9	**43.5**	14.8	43.0	44.1	61.5
CT45	53	C	I	1	**28.3**	**52.1**	29.1	63.5	**14.6**	36.3	29.9	61.6
CT46	56	C	III	1	**42.6**	**61.7**	39.5	29.2	52.3	31.3	38.9	48.3
CT47	31	E	III	3	**68.9**	17.8	38.3	**20.6**	10.1	30.8	56.7	38.0
CT48	52	C	II	2	42.0	53.2	38.7	**40.0**	**19.8**	56.4	59.8	52.8
CT49	63	S	III c	1	**56.9**	50.5	75.2	**35.7**	21.4	76.1	30.2	50.9
CT50	62	C	IV	1	**46.4**	**26.3**	44.7	39.7	35.0	68.5	36.8	54.3
CT51	67	C	III b	2	97.0	64.4	87.9	38.2	**22.3**	28.0	42.4	33.4
CT52	46	M	I a	1	**65.6**	47.5	42.6	45.2	81.1	34.6	**30.6**	74.2
CT53	40	S	III c	2	**58.5**	**23.1**	5.7	**49.7**	17.4	56.0	29.8	52.3
CT54	56	C	III c	1	28.6	**23.5**	13.7	39.4	**24.4**	23.4	**54.6**	54.0
CT55	66	M	I a	1	14.2	**8.3**	73.3	24.5	22.1	42.0	**33.8**	13.4
CT56	51	S	I c	1	**26.3**	**25.2**	87.0	**39.9**	16.9	25.1	17.8	31.3
CT57	55	S	I c	1	25.9	23.6	46.1	34.4	33.1	46.4	**57.9**	51.3
CT58	71	S	III c	3	**41.5**	69.9	50.4	**45.2**	31.9	32.8	33.8	41.0
CT59	53	S	II	2	29.0	25.1	38.1	**28.4**	29.6	46.1	**75.9**	37.8
CT60	55	E	I c	1	**66.2**	37.2	39.9	**37.9**	38.8	37.8	**49.4**	40.7
CT61	57	S	III c	2	90.4	22.6	25.9	26.0	37.2	28.5	38.6	33.9
CT62	48	M	II	1	34.6	24.7	33.7	**88.1**	49.9	37.4	27.3	46.3
CT63	73	M	III b	1	27.7	**75.0**	33.6	**39.8**	43.8	46.1	44.5	30.9
CT64	56	S	III c	1	**26.2**	**24.4**	44.8	**41.9**	30.7	42.1	52.6	38.3
CT65	51	S	IV	2	34.3	**38.3**	50.8	**53.4**	36.3	34.9	38.7	36.9
CT66	38	M	III c	2	**36.1**	23.5	47.9	41.8	32.1	37.1	36.9	27.1
CT67	42	E	I a	2	**37.6**	**33.6**	39.0	**54.1**	**49.9**	32.7	**41.1**	45.3
CT68	76	S	II c	3	30.5	**62.0**	50.5	34.3	55.2	38.7	35.3	32.7
CT69	72	C	I a	1	66.1	**35.4**	36.1	**27.2**	28.5	38.1	33.2	39.1
CT70	42	S	III c	3	40.4	30.2	50.5	44.1	35.7	48.6	39.7	32.7
CT71	50	S	III c	2	**56.6**	**48.9**	24.8	41.6	48.0	78.5	40.7	39.6
CT72	47	S	III c	2	44.3	**48.3**	33.9	35.4	34.6	37.1	56.5	25.9
CT73	41	S	II c	2	**67.8**	**23.4**	26.3	**56.3**	34.2	24.8	38.3	36.9
CT74	43	S	III c	2	**38.9**	**58.2**	33.4	35.4	34.6	37.3	56.5	25.9
