# Supplementary material for: High-throughput detection of aberrant imprint methylation in the ovarian cancer by the bisulphite PCR-Luminex method
Source: BMC Med Genomics. 2012 Mar 26;5:8. doi: 10.1186/1755-8794-5-8 (PMC3342152; doi:10.1186/1755-8794-5-8)
Supplement: Supplementary file 3 [file 1755-8794-5-8-S3.DOC]

Supplemental Table 2. Sequences of PCR primers and restriction enzymes used for PCR-RFLP analysis.

Gene	Target	Primer	Primer sequence (5'-3')	Amplicon (bp)	annealing	Accession No.	Enzyme	Reference
H19	gDNA	H19 RT F1<br>H19 RT R2	TGCTGCACTTTACAACCACTG<br>GTCGGAGCTTCCAGACTAGG	635	66	AF125183	*Rsa* I	Vambergue et al. 2007
	cDNA	H19 RT F1<br>H19 RT R2	TGCTGCACTTTACAACCACTG<br>GTCGGAGCTTCCAGACTAGG	554	66			
IGF2	gDNA/cDNA	IGF-II IP2<br>IGF-II IP3	TTGAGTCAAATTGGCCTGGA<br>TGCCAATTACATTTCATTTGC	279	60	AF517226	*Apa* I	Ogawa et al. 1993
KCNQ1	gDNA/cDNA	KCNQ1-14-F1<br>KCNQ1-14-R1	GGAGGAGAAGTGATGCGTGTCTT<br>GCACCGCTGATCATGCAGCTG	207	64	NC000011	*SmaI*	—
LIT1	gDNA	LIT1 SMS2-B<br>LIT1 SMS2-C	AGGAAAGTGTTGAGTGGTAA<br>CAAGCAGTACTGTTTCAGAA	803	60	NR002728	*Rsa* I	Higashimoto et al. 2000
	cDNA	Lit102<br>Lit202	TCTGGTTCATGTCACTCTGTGGAGCAG<br>CTCCCAAAAGCAGAGTTTTGGCAATAT	477	50			Mitsuya et al. 1999
GTL2	gDNA/cDNA	GTL2 F6<br>GTL2 R2	GTGTGTACCTTGGTTGGTGACTC<br>GAGGCATATATTTGAGTTACACATACCCCTTAGTCC	368	66	AF052114	*Taa* I	Wylie et al. 2000
PEG1	gDNA/cDNA	PEG1 PegF<br>PEG1 PegR	TACTAAACCAGCATACCCTTAC<br>GCAGTCATCATAAAGGAATCAG	308	66	AB045582	*Afl* III	Pedersen et al. 1999
PEG3	gDNA/cDNA	P3-MnlI-F<br>P3-MnlI-R	CCCTCCCCTCGCATAATAACTA<br>TCTTCTGTCTGTCTCCTCTCCC	339	60	AC006115	*Mnl* I	Maegawa et al. 2001
NDN	gDNA/cDNA	NDN NEC20F<br>NDN NEC6R	GCCCGAATACGAGTTCTTTT<br>CACACATCATCAGTCCCATA	540	66	NM002487	*Mbo* I	MacDonald et al. 1997
