# Supplementary material for: High-throughput detection of aberrant imprint methylation in the ovarian cancer by the bisulphite PCR-Luminex method
Source: BMC Med Genomics. 2012 Mar 26;5:8. doi: 10.1186/1755-8794-5-8 (PMC3342152; doi:10.1186/1755-8794-5-8)
Supplement: Supplementary file 4 [file 1755-8794-5-8-S4.DOC]

Supplemental Table 3. The list of LOH, LOI and MOI in HOC.

sample number	H19	IGF2	KCNQ1	LIT1	GTL2	PEG1	PEG3	NDN
NC1	-	MOI	-	MOI	-	-	-	-
NC2	-	LOI	-	-	MOI	-	-	-
NC3	MOI	MOI	MOI	-	-	MOI	-	-
NC4	-	-	-	MOI	-	-	-	-
NT1	-	-	-	MOI	-	-	-	-
NT2	MOI	MOI	MOI	-	-	-	-	-
NT3	-	-	MOI	-	MOI	-	-	-
NT4	-	-	-	-	MOI	MOI	-	-
NT5	MOI	-	-	MOI	MOI	MOI	-	MOI
NT6	MOI	MOI	-	-	MOI	-	-	-
NT7	-	-	-	MOI	MOI	-	-	-
CC1	MOI	-	-	-	-	MOI	-	-
CC2	-	-	-	-	-	-	MOI	MOI
CC3	-	-	-	-	-	MOI	-	-
CC4	-	-	MOI	-	-	-	MOI	-
CC5	-	-	MOI	-	-	-	-	MOI
CC6	-	-	LOI	MOI	-	-	-	-
CC7	-	-	MOI	-	-	-	-	-
CC8	-	LOI	MOI	-	-	MOI	-	MOI
CC9	-	-	-	-	MOI	MOI	MOI	-
CC10	-	-	-	-	MOI	-	-	-
CC11	-	-	-	-	-	-	-	-
CC12	-	-	-	-	-	MOI	MOI	MOI
CC13	MOI	MOI	LOI	-	MOI	MOI	MOI	-
CC14	-	-	-	-	-	-	-	-
CC15	-	-	-	-	-	LOI	-	MOI
CC16	-	-	-	-	-	-	-	-
CC17	-	-	-	MOI	-	LOI	-	-
CC18	MOI	-	-	-	-	-	-	-
CC19	-	-	-	LOI	-	-	-	-
CC20	-	LOI	-	-	LOI	LOI	-	-
CC21	MOI	-	LOI	-	LOI	-	-	-
CT1	-	LOH	MOI	-	MOI	-	-	-
CT2	-	-	MOI	-	-	-	-	-
CT3	LOI	-	MOI	MOI	MOI	-	-	MOI
CT4	-	-	LOI	-	LOH	-	-	-
CT5	-	-	-	-	-	-	-	MOI
CT6	-	-	MOI	-	-	-	-	MOI
CT7	-	-	-	-	LOI	-	-	-
CT8	-	-	LOI	LOI	LOI	-	-	MOI
CT9	-	-	-	-	MOI	-	MOI	MOI
CT10	LOH	LOI	MOI	-	-	-	-	-
CT11	MOI	-	-	-	MOI	LOI	-	-
CT12	ND	ND	LOH	-	MOI	-	MOI	-
CT13	LOI	LOI	-	-	-	MOI	MOI	-
CT14	-	MOI	MOI	-	-	-	-	MOI
CT15	ND	-	MOI	-	-	MOI	-	MOI
CT16	-	LOI	LOI	-	-	-	LOI	-
CT17	MOI	ND	-	-	MOI	LOI	MOI	-
CT18	-	-	MOI	MOI	MOI	-	MOI	-
CT19	MOI	-	MOI	MOI	LOI	MOI	MOI	-
CT20	MOI	-	MOI	-	MOI	LOI	MOI	LOI
CT21	LOI	ND	-	-	LOI	MOI	MOI	MOI
CT22	-	LOI	MOI	-	LOI	LOI	MOI	MOI
CT23	MOI	MOI	-	-	-	LOI	-	MOI
CT24	LOI	-	MOI	-	MOI	-	-	MOI
CT25	-	-	-	-	LOI	MOI	MOI	-
CT26	-	LOH	MOI	-	-	LOI	-	-
CT27	MOI	-	MOI	MOI	-	-	MOI	MOI
CT28	MOI	-	MOI	-	MOI	-	MOI	-
CT29	LOI	LOH	MOI	-	-	-	-	MOI
CT30	MOI	-	MOI	-	-	MOI	MOI	-
CT31	LOI	-	-	-	-	-	-	-
CT32	-	-	-	LOI	LOI	MOI	MOI	MOI
CT33	MOI	-	-	-	MOI	LOI	-	-
CT34	MOI	-	-	-	-	-	-	-
CT35	-	-	-	-	-	-	-	-
CT36	-	-	-	-	-	LOI	-	MOI
CT37	-	MOI	MOI	-	-	LOH	-	MOI
CT38	ND	LOI	LOI	-	MOI	LOI	MOI	-
CT39	LOI	LOI	-	-	MOI	MOI	-	-
CT40	LOI	-	-	-	-	MOI	-	MOI
CT41	-	LOI	-	-	-	LOI	MOI	-
CT42	MOI	-	-	-	MOI	-	-	-
CT43	-	MOI	-	-	MOI	LOI	ND	MOI
CT44	LOH	-	-	-	-	MOI	-	-
CT45	MOI	LOI	MOI	MOI	MOI	-	-	MOI
CT46	MOI	MOI	-	-	LOH	-	-	MOI
CT47	LOI	LOI	MOI	-	-	MOI	-	-
CT48	-	-	MOI	MOI	-	LOI	-	MOI
CT49	MOI	ND	MOI	-	-	MOI	-	MOI
CT50	MOI	MOI	-	-	MOI	-	-	-
CT51	-	-	-	MOI	-	-	-	-
CT52	LOI	LOI	LOI	-	-	-	LOH	-
CT53	MOI	-	MOI	-	MOI	LOI	-	-
CT54	-	-	-	MOI	MOI	-	MOI	MOI
CT55	-	-	MOI	-	MOI	-	MOI	MOI
CT56	MOI	-	-	-	MOI	MOI	-	-
CT57	-	-	-	-	-	-	MOI	LOI
CT58	MOI	-	-	-	ND	MOI	-	MOI
CT59	-	MOI	MOI	-	-	LOI	LOI	MOI
CT60	LOI	LOI	ND	-	-	MOI	MOI	-
CT61	-	-	MOI	-	-	-	-	-
CT62	-	LOI	MOI	-	-	LOH	-	-
CT63	-	-	-	-	LOH	LOI	-	-
CT64	MOI	MOI	MOI	-	MOI	MOI	-	-
CT65	-	LOI	MOI	-	MOI	LOH	-	MOI
CT66	MOI	MOI	ND	-	-	-	-	-
CT67	MOI	-	-	MOI	MOI	LOI	MOI	-
CT68	-	-	LOH	-	LOI	-	-	-
CT69	-	LOI	-	-	MOI	MOI	-	-
CT70	-	-	MOI	-	-	-	-	MOI
CT71	MOI	MOI	-	-	MOI	-	-	-
CT72	ND	MOI	-	-	LOI	-	-	-
CT73	LOI	LOI	MOI	-	MOI	LOI	-	MOI
CT74	MOI	-	-	-	LOI	-	-	-
